# Autologous temporomandibular joint reconstruction independent of exogenous additives: a proof-of-concept study for guided self-generation

**DOI:** 10.1038/srep37904

**Published:** 2016-11-28

**Authors:** Jiao Wei, Tanja Herrler, Dong Han, Kai Liu, Rulin Huang, Markus Guba, Chuanchang Dai, Qingfeng Li

**Affiliations:** 1 Department of Plastic and Reconstructive Surgery, Shanghai Ninth People’s Hospital, Shanghai Jiao Tong University School of Medicine, Shanghai, China; 2 Plastic Surgery and Burn Center, Trauma Center Murnau, Munich, Germany; 3 Klinik für Allgemeine, Viszeral-, Transplantations-, Gefäß- und Thoraxchirurgie, Klinikum der Universität München, Ludwig-Maximilians-Universität, Munich, Germany

## Abstract

Joint defects are complex and difficult to reconstruct. By exploiting the body’s own regenerative capacity, we aimed to individually generate anatomically precise neo-tissue constructs for autologous joint reconstruction without using any exogenous additives. In a goat model, CT scans of the mandibular condyle including articular surface and a large portion of the ascending ramus were processed using computer-aided design and manufacturing. A corresponding hydroxylapatite negative mold was printed in 3D and temporarily embedded into the transition zone of costal periosteum and perichondrium. A demineralized bone matrix scaffold implanted on the contralateral side served as control. Neo-tissue constructs obtained by guided self-generation exhibited accurate configuration, robust vascularization, biomechanical stability, and function. After autologous replacement surgery, the constructs showed stable results with similar anatomical, histological, and functional findings compared to native controls. Further studies are required to assess long-term outcome and possible extensions to other further applications. The absence of exogenous cells, growth factors, and scaffolds may facilitate clinical translation of this approach.

Joint defects from acute and chronic injury are common and often associated with impaired quality of life. Moreover, therapeutic management represents a significant burden for health systems worldwide. Currently, reconstructive surgery largely relies on the implantation of artificial joints. In clinical practice joint reconstruction based on allogenic donors and autologous substitutes is complicated due to shortage of allogenic donors/autologous donor sites, immune rejection and infection. Tissue engineered grafts are promising alternative options, but often exhibit insufficient biomechanical function, vascularization, and host-integration, as well as unknown risks associated with the use of exogenous cells, growth factors, and scaffolds^1^,^2^,^3^,^4^,^5^,^6^,^7^,^8^.

The temporomandibular joint (TMJ) features a particularly complex and precise joint configuration. Up to 25% of the population worldwide suffers from a TMJ-related medical condition that may severely affect physiological functions of daily life^9^,^10^. However, tissue engineering for articular reconstruction is still at an early stage of development^1^,^4^,^5^. In limited cartilage defects autologous chondrocyte implantation has been clinically applied for several years already. In contrast, the translation of anatomically shaped constructs for functional joint repair is still difficult to achieve^11^,^12^,^13^,^14^. Recent pioneering work has demonstrated the successful construction of customized anatomically shaped cartilage using human adipose-derived stem cells for total joint resurfacing^15^. In another ground-breaking study functional anatomically shaped cartilage tissue was created *in vitro* by mimicking the physiological process of mesenchymal condensation^16^. These *in vitro* findings remain to be tested *in vivo* regarding feasibility, bio-functionality and long-term outcome.

In the past years, scaffolds have been used either alone or in combination with exogenous cells and/or growth factors to promote the regenerative process, but the generation of composite tissue remains challenging. The recent trend from *in vitro* to *in vivo* generated tissue constructs emphasizes the importance of the *in vivo* microenvironment in the context of self-renewal^2^,^3^,^9^,^10^,^17^,^18^,^19^,^20^,^21^,^22^,^23^,^24^,^25^,^26^,^27^.

Here we report for the first time the *in vivo* self-generation of anatomically accurate chondro-osseous neo-tissue constructs consisting of bone and an articular cartilage surface absolutely independent of exogenous cells, scaffolds, and growth factors. We describe the method of guided generation, biomechanical properties, and preclinical results of autologous TMJ reconstruction in a goat model.

## Results

### Composite neo-tissue self-generation setup

**Figure 1:**
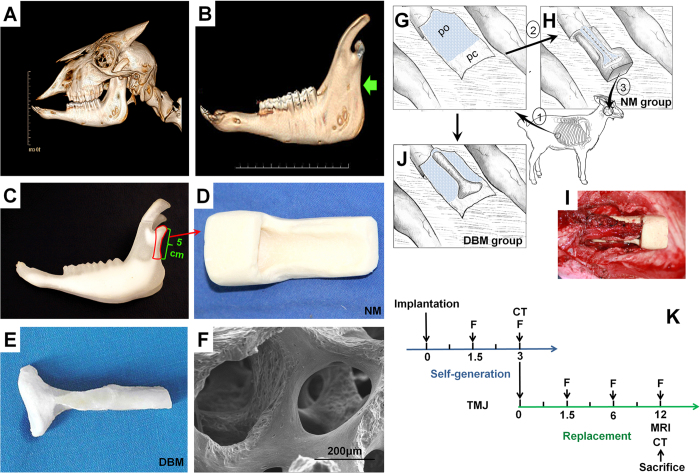
Preparation of the temporomandibular joint negative mold (NM) for the guided self-generation procedure in a goat model. (**A**) Based on cranial 3D CT, (**B**) the mandible was reconstructed and (**C**) printed in 3D. (**D**) A hydroxylapatite NM of the TMJ and mandibular ramus, 5 cm in length, was obtained. (**E**) After removal of a segment of the 3rd or 4th rib and costicartilage counting from caudally (#1) the regenerative unit was prepared from costal perichondrium and periosteum (#2). (**F**) Schematic illustration and (**G**) intraoperative view of the NM-guided tissue self-generation chamber including the previously prepared periosteum/perichondrium piece in combination with the NM. Finally, the self-generated tissue was used for autologous replacement in the temporomandibular joint (#3). (**H**) In the control group, the TMJ/mandibular ramus DBM scaffold was wrapped with the previously prepared periosteum/perichondrium. In both groups, the perichondrium covered the designated articular surface and the periosteum enclosed the osseous part. (**I**) An allogenic DBM scaffold of the same size served as positive control which was examined for (**J**) microstructure and porosity by scanning electron microscopy. (**K**) The study timeline consisted of 2 stages, construct self-generation and replacement, during which fluorescent labeling of osteogenesis, radiologic, histologic, and immunohistochemical analyses were performed. NM: negative mold; DBM: demineralized bone matrix; pc: perichondrium; po: periosteum; F: fluorescent labeling.

A hydroxylapatite (HA) negative mold (NM) of the mandibular condyle and adjacent ascending ramus portion was created based on cranial 3D CT reconstruction and 3D printing of the mandible in a goat model (Fig. 1A–D). A segment of the 3rd or 4th rib and costicartilage counting from caudally was removed as seen in schematic illustration and intraoperative view (Fig. 1E–G). The regenerative unit consisted of the NM in combination with the dissected costal perichondrium and periosteum. A demineralized bone matrix (DBM) scaffold derived from an allogenic mandible portionof the same size and region was examined for microstructure and porosity by scanning electron microscopy and wrapped with the previously prepared periosteum/perichondrium as positive control (Fig. 1H–J). In both groups, the perichondrium covered the designated articular surface and the periosteum enclosed the osseous part. The study timeline consisted of 2 stages, construct self-generation and replacement, during which fluorescent labeling of osteogenesis, radiologic, histologic, and immunohistochemical analyses were performed (Fig. 1K).

### Guided self-generation of chondro-osseous neo-tissue constructs

**Figure 2:**
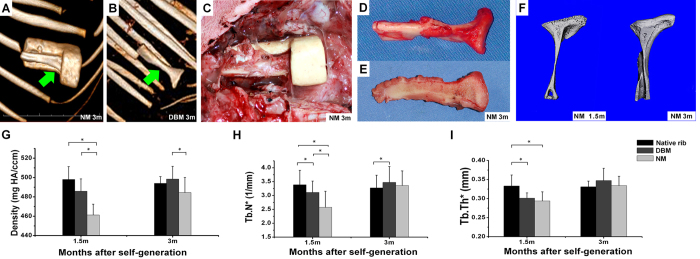
Guided self-generation of autologous neo-tissue. In both groups, (**A**) NM and (**B**) DBM, bone formation was observed using 3D examination at 3 months. (**C**) The intraoperative photograph of the NM chamber shows the generated construct within the NM chamber. (**D**) After NM removal, the neo-tissue unit exhibits precise anatomical configuration (**E**) and, in longitudinal section, cortical and cancellous bone, as well as a chondro-osseus transition zone. (**F**) Bone surfaces in the NM group were rough at 1.5 months, but smoothened over time as seen in micro-CT analysis. (**G**) In both groups, mineral density, (**H**) trabecular number, and (**I**) thickness of the neo-tissue increased from 1.5 to 3 months, no significant differences were found after 3 months compared to native rib. NM: negative mold; DBM: demineralized bone matrix; Tb.N: trabecular number; Tb.Th: trabecular thickness.

In both groups, negative mold and allogenic DBM (Fig. 2A,B), neo-bone formation was observed using 3D CT. At harvest, the generated construct was found aligned to the negative mold and, after negative mold removal, exhibited the precise shape of the positive resin model (Fig. 2C,D). Longitudinal sections of the newly formed construct demonstrated the existence of cortical and cancellous bone structures and a chondro-osseous transition zone (Fig. 2E). Cartilage thickness was slightly increased in both groups compared to native TMJ. Micro-CT analysis showed that newly formed bone surfaces in the negative mold group were rough with a slight irregular shape at 1.5 months (Fig. 2F). Moreover, cancellous and cortical bone boundaries were less distinct compared to the DBM group. However, rough surfaces smoothened over time and no significant differences were seen after 3 months (Fig. 2F). Mineral density, trabecular number, and neo-tissue thickness (Fig. 2G,H,I) in both groups increased during follow-up, whereas at 3 months, no significant differences were found compared to native rib (Fig. 2G,H,I).

### Histological work-up of generated neo-tissue constructs

**Figure 3:**
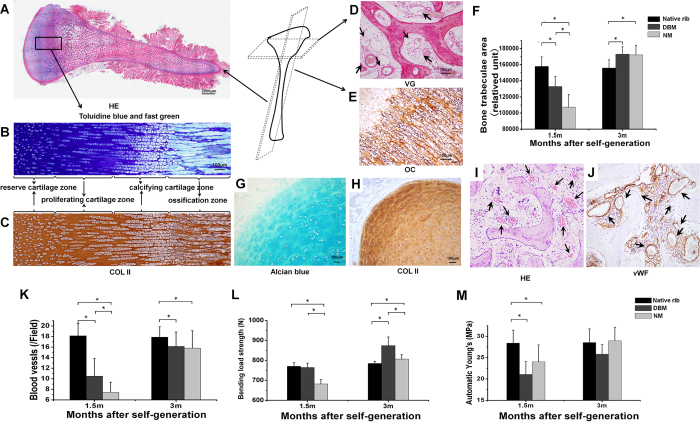
Histological and immunohistochemical analysis of the neo-tissue after 3 months of NM-guided self-generation. (**A**) HE, (**B**) toluidine blue and fast green, and (**C**) immunohistological staining against Col II showed four typical zones of chondro-osseous transition. (**D**) In VG and (**E**) immunohistochemical OC labeling, typical bone tissue structure was observed. (**F**) Histomorphometrically, trabecular areas were initially reduced in both DBM and NM groups compared to native rib, but significantly increased 3 months postoperatively. Hyaline cartilage tissue stained with (**G**) Alcian blue and (**H**) against Col II showed typical hyaline cartilage tissue architecture. Signs of angiogenesis (black arrows) were observed in both groups as seen in (**I**) HE and (**J**) immunohistochemical vWF. (**K**) Despite the increase in newly formed blood vessels between 1.5 and 3 months in both groups, their number was still significantly lower compared to native rib. (**L**) Bending load strength in the NM group was similar with native rib after 3 months, while this biomechanical parameter rapidly increased in the DBM group. (**M**) Young’s modulus increased in both groups over time with no significant difference after 3 months. HE: haematoxylin and eosin; Col II: collagen II; VG: Van Gieson; OC: osteocalcin; NM: negative mold; DBM: demineralized bone matrix; vWF: Von Willebrand Factor.

Longitudinal sections of the negative mold-guided condyloid process with articular cartilage surface and portion of the ascending mandibular ramus showed four typical zones of chondro-osseous transition consisting of reserve cartilage, proliferating cartilage, calcifying cartilage, and ossification at 3 months based on haematoxylin and eosin (HE), toluidine blue and fast green, and immunohistological anti-collagen II (Col II) staining (Fig. 3A,B,C). During early stages, chondrocyte size was larger and arrangement sparser as compared to native costicartilage, while after 3 months no significant difference was found. Histological Van Gieson (VG) and immunohistochemical anti-osteocalcin (OC) staining exhibited typical bone tissue structure including osteoblasts, trabecula, bone marrow cavity, cortical and cancellous bone (Fig. 3D,E). As to histomorphometrical results, trabecular areas were initially reduced in the DBM group and even more so in the negative mold group compared to native rib, but were significantly increased 3 months postoperatively (Fig. 3F). The hyaline cartilage tissue staining using Alcian blue and immunohistochemical staining against Col II (Fig. G,H) clearly showed typical hyaline chondrocytes, cartilage lacuna and matrix. Signs of angiogenesis were found in both groups as seen in HE, VG, and immunohistochemical Von Willebrand factor (vWF) (Fig. 3I,D,J) staining. The number of newly formed blood vessels considerably increased between 1.5 and 3 months in both groups, but was still significantly lower compared to native rib (Fig. 3K). As to biomechanical parameters, bending load strength in the negative mold group at 1.5 months was significantly lower compared to DBM and native rib, but increased to levels of native rib after 3 months. By contrast, DBM bending load strength was considerably increased compared to native rib (Fig. 3L). Young’s modulus showed lower values in both negative mold and DBM groups compared to native rib at 1.5 months, although the difference was not significant for negative mold. After 3 months no significant difference was found between the three groups.

### Application of the generated neo-tissue in temporomandibular joint repair

**Figure 4:**
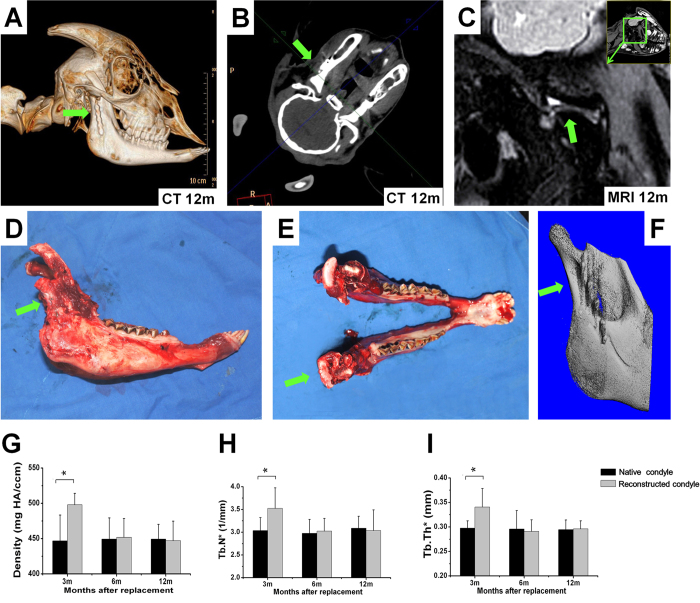
TMJ reconstruction stage. Based on (**A**) cranial 3D CT and (**B**) CT images the neo-tissue construct was fully integrated into the mandibular recipient site with anatomically correct articulation 12 months after replacement. (**C**) In MRI examination smooth articular surface of the reconstructed condyle was seen. (**D**) Lateral and (**E**) cranial views showed symmetric growth of the reconstructed area compared to the contralateral intact side. After initially rapidincrease in vascularization, mineral density, trabecular number and trabecular thickness the generated bone exhibited regular bone characteristics at 6 and 12 months as confirmed by (**F**) micro-CT results, (**G**) mineral density, (**H**) trabecular number, and (**I**) trabecular thickness levels in comparison to native bone. Tb.N: trabecular number; Tb.Th: trabecular thickness.

At 12 months after implantation of the self-generated neo-tissue construct for TMJ reconstruction, cranial 3D CT and CT images (Fig. 4A,B) showed full integration of the replaced structure into the mandibular recipient site. The reconstructed mandibular condyle articulated with the glenoid fossa in an anatomically correct position with a smooth articular surface except for one case of irregular surface and fibrosis as seen in MRI (Fig. 4C). Follow-up examinations showed that the reconstructed mandibular ramus exhibited symmetric growth compared to the contralateral intact side (Fig. 4D,E). Micro-CT (Fig. 4F) and calculated results including mineral density, trabecular number, and trabecular thickness (Fig. 4G,H,I) showed that the newly formed bone underwent a process of rapid increase in these parameters at 3 months postoperatively which returned to normal levels by 6 months and remained stable after 12 months.

### Structural and functional characteristics of the neo-joint after replacement

**Figure 5:**
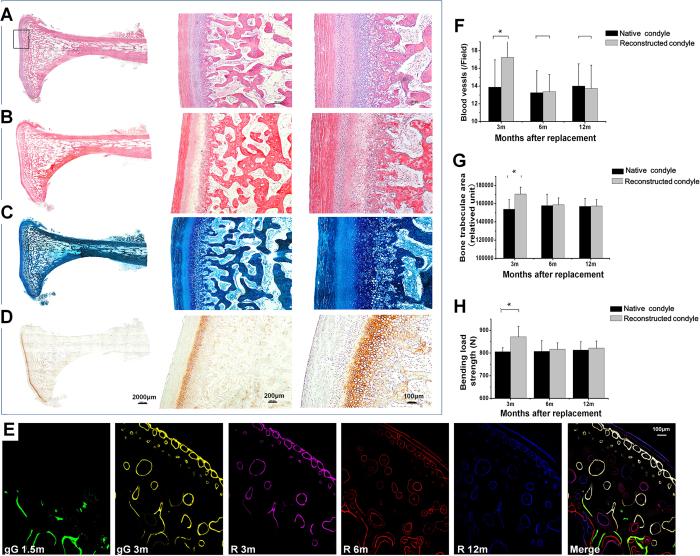
Histological and immunohistological analysis of the generated neo-tissue. Similar cartilage thickness was seen in histological sections stained with (**A**) HE, (**B**) VG, (**C**) toluidine blue and fast green staining, and (**D**) immunohistochemically against Col II compared to contralateral control 12 months after replacement. (**E**) New bone formation and centripetal ossification from the subperiosteum were identified based on merged real-time images from fluorescent labeling of osteogenesis (green: calcein; yellow: hydrochloride tetracycline; pink: xylenolorange; red: alizarin red; blue: calcein blue) during guided self-generation (1.5 and 3 months) and reconstruction stages (3, 6, and 12 months). Following a significant increase in (**F**) number of vessels, (**G**) trabecular areas, and (**H**) bending load strength of the replaced mandibular ramus compared to the contralateral native side 3 months after replacement, no significant differences were seen after 12 months. gG: guided self-generation stage; R: replacement stage.

Histological sections of the generated condyloid process with articular surface and large portion of the ascending mandibular ramus stained with HE, VG, toluidine blue and fast green, and immunohistochemically against Col II (Fig. 5A–D) showed similar cartilage thickness at 12 months compared to the contralateral side. The merged osteogenesis real-time images based on transmission microscope slices during guided generation and replacement stages showed new bone formation and a centripetal ossification pattern emerging from the subperiosteum (Fig. 5E). The number of vessels, trabecular areas, and bending load strength (Fig. 5F,G,H) in the replaced mandibular ramus were significantly increased compared to the contralateral native side at 3 months postoperatively, whereas no significant difference was found regarding these parameters after 12 months. Lateral range of motion of the mandible was 16 ± 1.2 mm and maximum mouth opening 34.9 ± 2.1 mm at 12 months postoperatively.

## Discussion

In this study we have shown the feasibility of guided self-generation of autologous tissue. This was achieved by mobilizing the *in vivo* microenvironment of the costal periosteum and perichondrium in combination with a negative mold. The generated neo-tissue units demonstrated promising biomorphological and mechanical features and, after implantation for autologous repair, sufficient *in vivo* functionality.

Periosteum and perichondrium are known to be essential for bone and cartilage generation *in vivo*^28^,^29^,^30^,^31^,^32^. Their cambium layer is rich in pluripotent cells which are able to differentiate into osteoprogenitor cells, chondroblasts, and osteoblasts. The results presented here showed the presence of hypertrophic chondrocytes around the cortical and cancellous bone in the subperiosteal space. This combination suggests endochondral ossification as mechanism of osteogenesis wherein the cartilage is remodeled to yield bony tissue. However, using fluorescent osteogenesis labeling we found a centripetal ossification pattern originating from the subperiosteum. This finding indicated that the periosteum was the source of newly formed tissue which is in line with previous reports of bone and cartilage regeneration^27^. Guided self-generated constructs underwent a process of transient rapid proliferation before returning to regular tissue characteristics. Compared to native controls, these ultimately demonstrated similar anatomical morphology, histology, vascularization, and, most importantly, optimal biomechanical properties. It is noteworthy that the transition zone of costal periosteum and perichondrium not only provided the ideal tissue types for joint reconstruction during the guided generation process, but was also exposed to constant respiratory mechanics which may have additional stimulatory impact on bone and cartilage formation.

The widely used DBM scaffold is known for its osteoinductive and osteoconductive effects^33^. Our results showed bony tissue characteristics. However, we were also able to distinguish cartilage and chondro-osseous transition on the uniformly structured DBM scaffold indicating the unique role of periosteum and perichondrium in cell differentiation and tissue generation. In fact, the DBM group showed enhanced osteogenesis, chondrogenesis, and angiogenesis. Since the scope of this study was to investigate the feasibility of *in vivo* tissue generation free of exogenous elements, only the negative mold group was pursued for TMJ repair, while the DBM group involving exogenous scaffold material only served as positive control during the generation stage.

The approach presented herein has enabled us to overcome obstacles that have impeded translation of tissue engineering into clinical practice. The guided self-generated tissue constructs exhibited accurate anatomical outline and genuine histological architecture. Moreover, biomechanical test results in goat demonstrated equivalent biomechanical and biological function compared to native tissue. After replacement, optimal osseointegration allowed for regular supporting and masticatory function. It is important to note that the generated neo-tissue exhibited robust vascularization ensuring immunological function. Exact shape and size of the generated construct were ensured by CAD/CAM technique for 3D printing of the personalized HA negative mold. Due to its material properties, i.e. non-biodegradable inertia and structural stability, temporary implantation of HA as a shaping guidance conformed to our requirements of autonomously generated tissue. The HA, indeed, showed no signs of degradation during the whole *in vivo* process. Moreover, its surface did not exhibit any porous structures allowing for easy removal of the generated tissue from the mold. Abandoning the use of exogenous additives not only avoids unknown risks and safety issues, but also accelerates translation into clinical practice.

Regardless of the multiple advantages, the guided generation process requires a long time period of several months and a multi-stage surgical procedure. In addition, patient age should be considered because of impaired regenerative capability due to aging. The present technique is currently limited by the size of costal periosteum and perichondrium. Periosteum/perichondrium in other parts of the body such as ilium, cranium, and tubular bones may be explored as possible sources for large-scale neo-tissue formation.

*In vivo* guided composite tissue generation independent of exogenous factors represents a significant development in the reconstruction of complex joints such as the TMJ. Further studies are required to assess clinical application, long-term biomechanical function, and potential extension of this technique to other complex anatomical structures. More autologous tissues with regenerative capacity may be identified in the future and combined with present translational strategies for the generation of composite tissues and complex organs.

## Materials and Methods

### Animals and surgical procedures

All animal procedures in this study were approved by Laboratory Animal Research Committee of Shanghai Jiao Tong University School of Medicine. All surgeries were performed under aseptic conditions. All experiments were performed in accordance with relevant guidelines and regulations.

### Negative mold preparation using CAD/CAM technique

A cranial 3D CT (120 kV, 70 mA, GE Medical Systems, Light-Speed16, New York) was performed with a slice thickness of 0.625 mm. The mandible was reconstructed and printed in 3D using a CAD/CAM system (Spectrum 510, Z Corporation). A positive resin model of the condyloid process and large portion of the ascending mandibular ramus with a length of 5 cm and a thickness of 1.5 cm was obtained. A hydroxylapatite (HA, Beierkang Medical Implants, Shanghai) negative mold was fabricated from the positive model^34^,^35^,^36^. Briefly, the HA powder was mixed with the liquid coagulation accelerator (both Beierkang Medical Implants, Shanghai) in a 2:3 ratio, poured into a container comprising the positive mold of the mandibular condyloid process and ascending ramus portion. Container and positive mold were removed after solidification to obtain the negative mold.

### Allogenic DBM scaffold preparation

DBM scaffolds serving as positive control were prepared as previously reported. Briefly, allogenic mandibles were harvested from which articular cartilage and soft tissue were completely removed while preserving the original shape. The mandibular condyle with articular cartilage surface/large portion of the ascending mandibular ramus was trimmed to a size of 5 cm × 1.5 cm equivalent to the printed positive resin model described above. After decellularization, demineralization, and defatting, DBM microstructure and porosity were examined by scanning electron microscopy (PhilipsXL-30, Amsterdam).

### Study design

A total of 30 healthy skeletally mature goats, 15 male and 15 female, age range 1–1.5 years, weighing 30 ± 5 kg were included in the study. In order to eliminate individual differences, each animal underwent negative mold-guided self-generation along with a contralateral DBM control, the sides were randomly assigned (Fig. 1K). In the self-generation stage, 6 goats were humanely euthanized at 1.5 and 3 months accounting for 12 individuals in total. All animals underwent fluorescent osteogenesis labeling and radiologic imaging. After being humanely sacrificed, specimens of self-generation and DBM controls were subjected to histological and biomechanical analysis.

During the replacement stage, 18 goats underwent reconstructive TMJ surgery using the self-generated neo-tissue construct which exhibited the anatomically accurate shape and configuration of the mandibular condyle/ascending mandibular ramus portion. Fluorescent osteogenesis labeling and radiologic imaging was performed on all animals. After 1.5, 6, and 12 months, 6 animals were humanely euthanized and subjected to analysis at each time point. Histological and biomechanical analysis was performed on the obtained specimens.

All surgical procedures in this study were approved by the Animal Care and Experimental Ethics Committee of Shanghai Jiaotong University Medicine School.

### Creation of the regenerative unit

After exposure of the cartilage bone transition zone of third or fourth rib counting from caudally, periosteum and perichondrium were incised along the curve of the rib. A segment of rib and costicartilage was removed to obtain periosteum of 5.5 cm and perichondrium of 2.5 cm in length which was dissected from the parietal pleura as one piece and moved to a subcutaneous position with the neurovascular bundle serving as pedicle.

### Negative mold group

The negative mold guided self-generation chamber was created using the previously prepared piece of periosteum/perichondrium in combination with the negative mold which was fixed subcutaneously with the cambium layer facing the chamber. The perichondrium covered the designated cartilaginous articular surface in the negative mold chamber, whereas the periosteum enclosed the osseous part of the corresponding mandibular condyle/large portion of the ascending mandibular ramus.

### DBM group

The mandibular ramus DBM scaffold was wrapped with the previously prepared periosteum/perichondrium sheet, the perichondrium covering the articular surface and the periosteum enclosing the osseous part. At 1.5 and 3 months after surgery six goats were humanely euthanized and the generated TMJs harvested for further analysis.

### TMJ replacement surgery

Following 3 months of guided generation, the negative mold was explanted and the neo-tissue consisting of bone and articular cartilage used for unilateral replacement of the mandibular condyloid process with articular cartilage surface and adjacent large portion of the ascending mandibular ramus as previously described. Specifically, the condylar part was removed and the posterolateral mandibular ascending branch was chiseled out of the bone cortex according to the size of the generated neo-tissue. After checking occlusion function, the construct was fixed to the mandibular branch using mini-plates and titanium screws (Stryker, Michigan). Care was taken to align posterior edge of the generated mandibular branch with the mandibular ascending branch, and that the generated condyle was positioned in the anatomically correct position in the articular fovea of the TMJ. Postoperatively, eating habit, maximum interincisal opening, jaw mobility, and occluding relation were examined to evaluate mandibular function. After 3, 6, and 12 months postoperatively six animals were sacrificed at each time point for radiological, histological, and biomechanical examination and compared with the contralateral native TMJ as control.

### Fluorescent labeling of osteogenesis

In the guided generation stage, all animals (n = 12) received i.m. injection of 25 mg/kg hydrochloride tetracycline, 20 mg/kg calcein 7 days before sample collection at 1.5 (n = 6) and 3 (n = 6) months. After replacement, 90 mg/kg xylenolorange, 25 mg/kg alizarin red, and 30 mg/kg calcein blue were applied for persistent osteogenesis labeling (all reagents from Sigma, USA) 7 days before sample collection at 3 (n = 6), 6 (n = 6), and 12 (n = 6) months^37^.

Samples were fixed and embedded in methylmethacrylate as previously described^36^,^37^. Sections of 100 μm were examined using a confocal laser scanning microscope (Leica TCS Sp2 AOBS, Germany) to identify mineralization deposition as an indicator of osteogenesis.

### Imaging analyses

During the guided generation stage, thoracic 3D CT was performed at 1.5 and 3 months postoperatively to observe *de novo* tissue formation. After replacement, the TMJ was examined using cranial 3D CT and magnetic resonance imaging (MRI) at 3, 6, and 12 months postoperatively to evaluate osteogenesis, chondrogenesis and joint function of the reconstructed TMJ and mandibular ramus. Micro-CT was performed on the generated mandibular ramus after harvest based on a previously reported protocol to examine bone formation. Microtomographic slices were acquired for structural assessment and calculation of morphometric data of osteogenesis including mineral density, volume, trabecular number, and thickness.

### Histological analysis of osteogenesis, chondrogenesis, and angiogenesis

Samples of generated bone, cartilage, and their transition zone were collected and processed during both guided generation and reconstruction stages. Sections of 5 μm were made and 20 slices randomly chosen from each sample for staining with HE, toluidine blue and fast green, VG, and Alcian blue to examine osteogenesis, chondrogenesis, and vascularization. Under a light microscope (Olympus, Japan), 10 fields were randomly chosen from each slice for software-based analysis (Origin 8.0) of trabecular bone areas and blood vessel density. Immunohistochemical assessment of osteogenesis, chondrogenesis, and vascularization was performed using OC, Col II and vWFantibodies (all from Abcam, USA) as previously described^36^.

### Biomechanical analyses

Biomechanical properties of the newly formed bone and cartilage of NM and DBM groups were assessed based on bending load strength and Young’s modulus in comparison to native tissue. Samples of the harvested tissue specimens of all groups, native, NM, and DBM, were stored at −80 °C prior to testing. Examinations were conducted using a universal material testing machine (Instron 8874, USA)according to a previously described protocol^38^. After thawing, bone and cartilage specimens were trimmed to obtain a similar size with planoparallel ends to take the compression test. For bending load strength, bone specimens were placed in a three-point bending system, providing an unsupported length of 1 cm. Both ends of the tested tissue were not fixed. Then, 3 points of the specimen were randomly chosen in a three-point bending system. Load was applied to the midpoint of the unsupported length. The assessment of Young’s modulus was performed by measuring the cartilage specimens using the strain gauge at a loading rate of 2 mm/min with persistent strength and stopped following cartilage deformation.

In the replacement stage, the bending load strength and Young’s modulus were performed as described above to assess the biomechanical properties of the reconstructed mandibular condyle compared to the contralateral native condyle.

### Statistical analysis

All results are presented as mean ± standard error. Statistical analysis was performed using one-way analysis of variance for comparison between the groups using SPSS software (SPSS, Inc). Significant differences were indicated as p < 0.05.

## Additional Information

**How to cite this article**: Wei, J. *et al*. Autologous temporomandibular joint reconstruction independent of exogenous additives: a proof-of-concept study for guided self-generation. *Sci. Rep.* **6**, 37904; doi: 10.1038/srep37904 (2016).

**Publisher's note:** Springer Nature remains neutral with regard to jurisdictional claims in published maps and institutional affiliations.
